# P2Y_12_ Antagonists in Cardiovascular Disease—Finding the Best Balance Between Preventing Ischemic Events and Causing Bleeding

**DOI:** 10.3389/fcvm.2022.854813

**Published:** 2022-05-12

**Authors:** Himawan Fernando, James D. McFadyen, Xiaowei Wang, James Shaw, Dion Stub, Karlheinz Peter

**Affiliations:** ^1^Atherothrombosis and Vascular Biology Laboratory, Baker Heart and Diabetes Institute, Melbourne, VIC, Australia; ^2^Department of Medicine, Monash University, Melbourne, VIC, Australia; ^3^Department of Cardiology, The Alfred Hospital, Melbourne, VIC, Australia; ^4^Department of Cardiometabolic Health, University of Melbourne, Parkville, VIC, Australia; ^5^Thrombosis and Hemostasis Unit, Department of Clinical Hematology, The Alfred Hospital, Melbourne, VIC, Australia; ^6^Department of Immunology, Monash University, Melbourne, VIC, Australia

**Keywords:** P2Y_12_ receptor, P2Y_12_ receptor antagonists, platelet receptors, antithrombotic therapy, myocardial infarction, percutaneous coronary intervention, high on treatment platelet reactivity

## Abstract

Dual antiplatelet therapy comprising of aspirin and oral P2Y_12_ receptor antagonists are an established cornerstone of therapy in acute coronary syndromes and percutaneous coronary intervention. As a result, the platelet P2Y_12_ receptor remains a key therapeutic target in cardiovascular medicine since pharmacological antagonists were first developed in the 1990’s. With a greater understanding of platelet biology and the role played by the P2Y_12_ receptor in the amplification of platelet activation and thrombus formation, there has been progressive refinement in the development of P2Y_12_ receptor antagonists with greater potency and consistency of antiplatelet effect. However, challenges remain in the utilization of these agents particularly in balancing the need for greater protection from ischemic events whilst minimizing the bleeding risk and present a real opportunity for the institution of individualized medicine. Future drug developments will provide clinicians with greater avenues to achieve this.

## Introduction

The platelet P2Y_12_ receptor has remained a key therapeutic target in cardiovascular medicine since the discovery of ticlopidine’s antiplatelet effects. This review will discuss the role of the P2Y_12_ receptor in platelet activation and explore the range of therapeutic agents inhibiting this receptor. It will focus particularly on exploring the current clinical challenges with respect to the ongoing goal of preventing ischemic cardiovascular events whilst avoiding bleeding complications. Lastly, we will look to future directions with respect to targeting the P2Y_12_ receptor including the role of reversal agents and the novel subcutaneous P2Y_12_ inhibitor, selatogrel.

## The Platelet P2Y_12_ Receptor and Its Role in Platelet Activation

Platelets are non-nucleated fragments, released from their parent cells, megakaryocytes, which reside in the bone marrow. Each platelet is 2–4 microns in diameter and has an average lifespan of 10 days (1). The exterior surface of the platelet contains an array of adhesion receptors and soluble agonist receptors which play critical roles in mediating platelet adhesion and activation (1).

Platelets circulate in a resting state, however, in the face of vascular injury, or exposure to thrombogenic surfaces such as a ruptured atherosclerotic plaque, platelets quickly adhere, activate and aggregate with a subset of platelets becoming highly activated (procoagulant platelets) and thus help amplify thrombin generation and fibrin formation.

In addition, activated platelets form heterotypic aggregates with leukocytes that act to enhance thrombus formation, amplify inflammation and the release of neutrophil extracellular traps (NETs) and have been implicated in atherothrombosis (2).

Platelet activation is a complex process involving multiple biochemical and biophysical signaling pathways and ultimately leads to an increase in intracellular calcium concentration that underpins multiple platelet functional responses critical to thrombus formation. These include platelet shape change, platelet degranulation and the release of prothrombotic soluble agonists such as adenosine diphosphate (ADP) and Thromboxane A2 (TXA2), the activation of the major platelet adhesion receptor, integrin GPIIb/IIIa (αIIbβ3, CD41/CD61), to adopt a high affinity for its major ligand, fibrinogen, and the expression of negatively charged phospholipids, such as phosphatidylserine on the platelet surface (1). The activation of platelets by soluble agonists such as ADP, TXA2, and thrombin are critical mediators of platelet activation. Soluble agonists mediate platelet activation *via* the activation of their respective cognate G protein coupled receptor (GCPR), which subsequently triggers second-messenger pathways (1).

The P2Y_12_ receptor, is a G protein coupled receptor (GPCR) that couples predominantly to the Gαi2 signaling family and is activated by ADP. Activation of the P2Y_12_ receptor mediates several important platelet functional responses. Indeed, P2Y_12_ activation potentiates platelet granule secretion and platelet TXA2 generation. Critically, P2Y_12_ activation linked PI3 kinase generation and subsequent downstream Rap1b activation plays a critical role in regulating the sustained activation of GPIIb/IIIa. As such, P2Y_12_ receptor function serves an important role in mediating platelet activation, platelet thrombus growth and stability *in vivo*. These features are underscored by *in vivo* studies of mice lacking platelet P2Y_12_ expression which form smaller and more unstable thrombi in response to vascular injury. In accordance, patients with defects of P2Y_12_ receptor function typically exhibit diminished platelet aggregation in response to ADP, impaired granule secretion and a bleeding phenotype (3).

It is important to note that platelets express another P2Y ADP receptor, the P2Y_1_ receptor, which in contrast to P2Y_12_, is linked to Gαq signaling, and whose activation triggers the release of intracellular calcium, platelet shape change and only weak, transient platelet aggregation (4). Thus, whilst initiating only weak platelet activation in response to ADP activation, the P2Y_1_ receptor plays an important complementary role to P2Y_12_, since full ADP induced platelet activation can only occur in the presence of both the P2Y_1_ and P2Y_12_ receptors.

**FIGURE 1:**
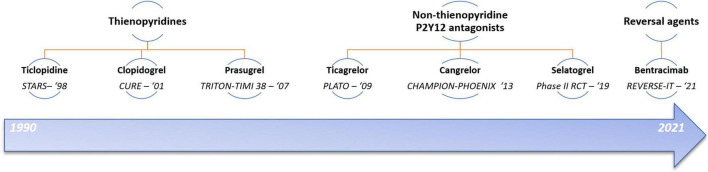
Temporal developments of P2Y_12_ receptor antagonists and associated landmark trials. Trials: STARS (12); CURE (5); TRITON-TIMI 38 (26); PLATO (25); CHAMPION-PHOENIX (112); Selatogrel—Storey et al. (109); REVERSE-IT (113).

ADP induced platelet activation is an important target for pharmacological antiplatelet therapies due to their central role in platelet activation (see Figure 1 for therapeutic developments related to the P2Y_12_ receptor) (5). In addition to antiplatelet effects, recent evidence has suggested that P2Y_12_ antagonists may have anti-inflammatory effects mediated by the indirect inhibition of platelet-leukocyte interactions or by the direct antagonism of P2Y_12_ receptors expressed on macrophages. Additionally, the P2Y_12_ receptor present on human cardiac-derived mesenchymal progenitor cells (hCPCs) resident in the adult heart appears to modulate the release of pro-survival exosomes which may provide protection against hypoxia induced cardiomyocyte apoptosis (6, 7). Ticagrelor appears to achieve this by increasing viable hCPCs, increasing extracellular adenosine and inhibiting the equilibrative nucleoside transporter 1 (ENT1), an adenosine transporter. However, clopidogrel which does not inhibit ENT1, does not appear to provide the same cardioprotective effects against hypoxia or the same increase in protective exosome levels in the presence of exogenous adenosine. Therefore, this beneficial effect appears to be unique to ticagrelor’s pharmacodynamic profile (6). Conversely, ticagrelor appears to inhibit harmful proinflammatory and prothrombotic platelet and leukocyte derived exosomes released during myocardial infarction compared to clopidogrel (8). This differential effect is postulated to be due to the greater potency of ticagrelor in P2Y_12_ receptor inhibition as reduced proinflammatory exosomes was also seen with prasugrel treatment compared to clopidogrel (9). It may also relate to ticagrelor’s preferential effects in increasing extracellular adenosine concentrations. These have been recognized as potential benefits of P2Y_12_ receptor antagonists (10).

## Past and Present Thienopyridine P2Y_12_ Antagonists

Interestingly, P2Y_12_ antagonists were developed before the human receptor had been sequenced in 2001 (11). Ticlopidine, originally developed in the search for an anti-inflammatory drug, was the first P2Y_12_ inhibitor available for clinical use in the early1990’s. This revolutionized percutaneous coronary intervention by providing much needed protection against coronary stent thrombosis when compared to anticoagulants such as warfarin, facilitating the increased utilization of these intracoronary devices (12). Much like subsequent thienopyridines, it was a prodrug requiring hepatic metabolism into active form. Thienopyridines bind covalently to cysteine residues of the P2Y_12_ receptor, modifying the binding site of ADP irreversibly for the life of the platelet (13). Due to a small but significant risk of potentially fatal blood dyscrasias at therapeutic doses of ticlopidine, it was promptly replaced by clopidogrel once available.

Clopidogrel was systematically developed using ticlopidine as a structural template with the aim of obtaining a strong anti-platelet effect without hematological side effects seen with ticlopidine. It was launched worldwide in 1998. After ingestion, clopidogrel is rapidly absorbed from the gastrointestinal tract, however, its absorption is influenced by the activity of the intestinal P-glycoprotein efflux transporter (14). Approximately 85% of the absorbed clopidogrel ester is inactivated by the hCE1 hepatic esterase (15). The remaining amount then requires two steps of hepatic metabolism from prodrug to active metabolite. The first step relies on hepatic cytochromes CYP2C19, CYP1A2, and CYP2B6 to the intermediate compound 2-Oxo-clopidogrel (16). This then undergoes a second stage of metabolism and oxidation through cytochromes CYP3A4, CYP2C19, CYP2B6, and CYP2C9 to its active metabolite. Clopidogrel’s active metabolite has a short half-life of 30 min (17). Formation of this active metabolite is impaired in patients with loss-of-function genetic polymorphisms, particularly involving the CYP2C19 allele (18).

Prasugrel is also a prodrug which is rapidly absorbed and completely hydrolyzed by the hCE2 intestinal esterase into an intermediate inactive metabolite (19). This then undergoes one hepatic step for conversion to its active form most reliant on cytochromes CYP3A4 and CYP2B6 (20, 21). Prasugrel’s active metabolite has a much longer half-life of 7 h compared to clopidogrel’s active metabolite (17). Both clopidogrel’s and prasugrel’s metabolites irreversibly bind and inhibit ADP binding to the platelet P2Y_12_ receptor.

Prasugrel is a more potent inhibitor of platelet reactivity compared to clopidogrel (17). It can achieve therapeutic antiplatelet effect at 2 h after loading dose whilst clopidogrel can take up to 4 h after a 600 mg loading dose. After a 60 mg loading dose, prasugrel demonstrated 91% inhibition of ADP-induced platelet aggregation at 2 h compared with only 69% inhibition with 600 mg of clopidogrel at 6 h (22). Interestingly, *in vitro* P2Y_12_ receptor binding affinity of clopidogrel and prasugrel appear to be the same suggesting that the greater antiplatelet effect of prasugrel is reliant on differences in pharmacokinetics. Prasugrel also produces a more consistent platelet inhibitory response with less inter-individual variability compared to clopidogrel. Furthermore, Heestermans and colleagues found that levels of platelet inhibition after 600 mg clopidogrel loading in ST elevation myocardial infarction (STEMI) were only approximately 7% after 4 h and 25% at 24 h compared to almost 60% at 6 h in healthy controls suggesting a much slower onset in myocardial infarction (23).

The antiplatelet effect of thienopyridines, such as prasugrel and clopidogrel, persist for the life of the platelet (24). Therefore normal platelet function returns approximately 7–10 days after cessation (21). As clopidogrel is a prodrug, a poor pharmacological response phenotype has been described where comorbidities, drug interactions and cytochrome P450 genetic polymorphisms lead to an impaired antiplatelet response (18). This appears to be less of an issue with prasugrel which also leads to more potent platelet aggregation and improved clinical outcomes compared to clopidogrel (18, 25, 26).

## Cyclopentyl-Triazolo-Pyramidine Agents and ATP Analogs

Ticagrelor belongs to the cyclopentyl-triazolo-pyrimidine class of P2Y_12_ receptor antagonist which binds reversibly to the P2Y_12_ receptor at a different binding site to the thienopyridines (clopidogrel and prasugrel) and ADP itself. Whilst the parent drug is in active form, it is nonetheless hepatically metabolized into an equally efficacious metabolite (AR-C124910XX) predominately through cytochrome CYP3A4 and CYP3A5 (21). This metabolite, AR-C124910XX has a half-life of approximately 8.5 h, whilst the parent compound has a half-life of 7 h (17).

Cangrelor is a direct acting, intravenous, reversible P2Y_12_ receptor antagonist designed to be an analog of adenosine triphosphate, an endogenous antagonist of the P2Y_12_ receptor. It does not require hepatic metabolism into active form and has a very short half-life of less than 9 min, reaching steady state within 30 min of infusion commencement when a bolus is given (21).

Cangrelor administered intravenously has the fastest onset of action with near complete inhibition of ADP induced platelet aggregation approximately 4 min after commencement of infusion (27). Ticagrelor like prasugrel, achieves therapeutic antiplatelet effect 2 h after loading dose and is a more potent inhibitor of platelet reactivity compared to clopidogrel (17). Ticagrelor achieved 88% inhibition of platelet aggregation after 180 mg loading dose at 2 h which is comparable to prasugrel. Ticagrelor and prasugrel also produce a more consistent platelet inhibitory response with less inter-individual variability compared to clopidogrel (28).

Due to the very short half-life and the reversible antagonism, platelet function is restored within 1 h of cessation of cangrelor infusion (17). In contrast, normal platelet function is only restored within 4–5 days of stopping ticagrelor compared to 7–10 days for clopidogrel and prasugrel (21).

## Clinical Application of P2Y_12_ Antagonists in Cardiovascular Diseases

Dual antiplatelet therapy (DAPT) is a well-established cornerstone of medical therapy for secondary prevention post-acute coronary syndrome (ACS), where it has been shown to reduce recurrent cardiovascular events (5, 29). DAPT consists of aspirin and P2Y_12_ receptor antagonists where the combined antiplatelet activity is superior to aspirin alone. Additionally, P2Y_12_ inhibitors play a pivotal role in reducing coronary stent thrombosis post percutaneous coronary intervention (PCI) (30, 31). Prasugrel and ticagrelor are the preferred agents in ACS based on current European (European Society of Cardiology—ESC) and American (American College of Cardiology—ACC) guidelines providing class I recommendations in these settings (32, 33). Clopidogrel remains the preferred agent in non-ACS settings and in specific scenarios, such as when a combination of antiplatelet and anticoagulation therapy is required (34).

## Challenges Relating to P2Y_12_ Receptor Antagonism

**FIGURE 2:**
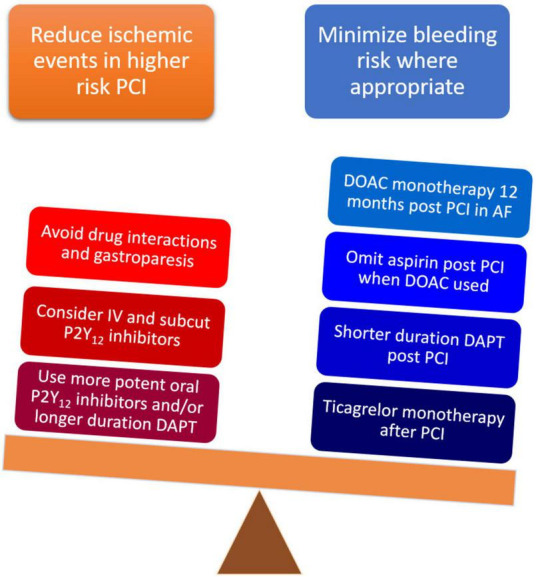
Major challenges related to the contemporary use of P2Y_12_ antagonists in clinical practice categorized by the two competing and inherently linked goals of reducing ischemic risk or minimizing bleeding risk.

There are several considerations in the clinical application of P2Y_12_ receptor antagonists. High on treatment platelet reactivity (HPR) has remained a persistent concern ever since the first use of these agents. Additionally, the search for the optimal regimen to balance bleeding risk with ischemic benefit continues (see Figure 2 for a summary of challenges relating to P2Y_12_ receptor inhibition) (35).

There is evidence suggesting that drug interactions or genetic polymorphisms that interfere with the pharmacokinetics of P2Y_12_ inhibitors may lead to poorer outcomes (18). Most notably, proton pump inhibitors were the focus of concerns regarding drug interactions with clopidogrel with studies suggesting a significant pharmacokinetic and pharmacodynamic interaction with observational clinical data suggesting worse outcomes with this combination (36–40). The mechanism of this interaction appeared to involve common cytochrome P450 pathways leading to impaired conversion of clopidogrel to its active form. Notably, with increasing clinical use of prasugrel and ticagrelor instead of clopidogrel, this interaction has become less relevant.

Concerns about the lack of sufficient therapeutic inhibition of platelet function has led to development of the concept of HPR. However, there is no consensus definition of HPR and its clinical relevance remains uncertain. Also arbitrary in their nature, some reference thresholds for HPR have been suggested for aspirin and P2Y_12_ inhibitors utilizing a variety of platelet function tests (41, 42). In the latest update, the following thresholds for HPR of P2Y_12_ inhibitors are recommended: VerifyNow > 208 platelet reactivity units, Multiplate Analyzer > 46 AU, thromboelastography > 47 mm and VASP-PRI assays ≥ 50% platelet reactivity index (43). However, these thresholds are all based on clopidogrel as there is no consensus agreement for prasugrel or ticagrelor and primarily for use in evaluating HPR in a research setting rather than for clinical practice (44). HPR has been associated with adverse cardiovascular events including death, AMI and stent thrombosis in observational studies which are prone to the influence of confounding factors (45). The incidence of HPR also varies considerably depending on the type of platelet function test used (46). Two prospective trials have also questioned the utility of platelet function testing in detecting HPR after ACS with respect to individualization of P2Y_12_ inhibition. In the ANTARCTIC study, use of platelet function testing to guide modification of the daily dose of prasugrel compared to a set dose of 5mg daily did not improve ischemic or bleeding outcomes post ACS (47). Similarly, in the ARCTIC study, patients scheduled for PCI were randomized to HPR guided P2Y_12_ inhibitor dose adjustment or conventional, fixed dose treatment (48). This regimen also included switching clopidogrel to prasugrel as one option in patients with HPR on clopidogrel and conversely, switching from prasugrel to clopidogrel if platelet reactivity was low on prasugrel to reduce bleeding risk. Again, no benefit in terms of ischemic or bleeding events was seen with this HPR guided approach.

However, other situations have been identified where delayed onset of action and impaired antiplatelet response remains an issue for all oral P2Y_12_ antagonists. It remains a serious concern in patients presenting with AMI, particularly STEMI and cardiogenic shock where it relates to multiple factors including delayed intestinal absorption, systemic vasoconstriction and hemodynamic instability (49–51). Alexopoulos and colleagues demonstrated that at 2 h post administration of ticagrelor and prasugrel, more than 30% of STEMI patients had HPR. This is very concerning as it suggests, 30% of patients do not have adequate antiplatelet protection at the time of primary percutaneous coronary intervention (PCI). This problem is compounded by the frequent use of morphine, which delays therapeutic platelet inhibition even further. Additionally, hypothermia in the setting of cardiogenic shock or cardiac arrest can also lead to HPR (52).

This is of significant concern as current ACC/ESC guidelines recommend primary PCI in the management of STEMI where mechanical reperfusion can be established within 90 min of first medical contact at a PCI-capable hospital or within 120 min taking into consideration transfer time to a PCI-capable hospital (32, 33). Hence the time taken for platelet reactivity to fall below HPR cut-offs is significantly longer than the recommended timeframe in which primary PCI is performed in STEMI. Early maximal platelet inhibition during this time when PCI is performed is the ideal scenario to minimize the risk of acute thrombotic complications. However, it is clear from the available evidence that orally administered P2Y_12_ inhibitors do not achieve this goal during primary PCI in STEMI and are even less likely to in the presence of opioids (49). In this scenario, unless inhibition of platelet aggregation is achieved using cangrelor or GP IIb/IIIa inhibitors, anticoagulants such as unfractionated heparin are relied upon to protect against thrombotic complications during PCI. This does not, however, obviate the need for adequate platelet inhibition post procedure given the short half-life of peri-procedural intravenous heparin (53).

Finally, opioids contribute to early HPR post loading doses of all oral P2Y_12_ inhibitors by delaying their intestinal absorption (54–56). This is an emerging field of research first prompted by the finding by Meine and colleagues of an association between IV morphine and a higher rate of death in patients with non-ST elevation myocardial infarction (NSTEMI), which remained significant after propensity score matching, compared to nitrate therapy (57). Following this, several biochemical studies suggested that both morphine and fentanyl impair the absorption of all oral P2Y_12_ inhibitors by demonstrating lower plasma concentrations of the active compound after oral loading dose administration. This correlated with early HPR in patients administered opioids compared to those that were not (54, 58, 59). The clinical significance has been difficult to elucidate as all existing trials are retrospective in nature and have reported conflicting results, although a possible signal to harm based on surrogate markers of infarct size has been identified (57, 60–64). As a result, the search for strategies to mitigate the interaction or alternative analgesics to opioids is underway (65). Use of a pro-kinetic such as metoclopramide or bridging with IV cangrelor may be an effective strategy (66). Alternatively, lidocaine and acetaminophen appear to be promising alternative analgesics to opioids that do not interact with oral P2Y_12_ inhibitors (67, 68). This will be an important area of future research in addressing early HPR in a high-risk population (69).

## Balancing Bleeding Risk and Ischemic Outcomes

The balance between bleeding and ischemic risk remains one of the major challenges with P2Y_12_ inhibitors and indeed antithrombotic agents as a class (70). There seems to be an inherent link, the greater the potency of the P2Y_12_ inhibitor the greater the risk of bleeding. This is a significant concern due to the clear and consistent link between major bleeding events and adverse cardiac outcomes including increased mortality post PCI and in ACS (71, 72). Balancing ischemic events vs. bleeding risks provides an important opportunity for individualized medicine, to tailor potency of P2Y_12_ inhibitor use, either alone or in combination with aspirin, for each individual and their respective ischemic and bleeding risk. Patients post PCI with greater procedural complexity or cardiovascular co-morbidities, particularly diabetes, may benefit from greater than 12 months and up to 36 months of DAPT achieved by continuing oral P2Y_12_ inhibition, although this is invariably associated with increased bleeding risk (73–77). In contrast, where PCI occurs in lower risk, straightforward procedures using the latest generation coronary stents, a shorter duration of DAPT followed by P2Y_12_ inhibitor monotherapy may be the optimal solution. A 3-month duration of DAPT followed by ticagrelor monotherapy has been shown in multiple trials to reduce bleeding risk without increasing ischemic risk (78–80).

An even shorter duration of only 1 month with DAPT followed by ticagrelor monotherapy has been studied in the MASTER-DAPT and GLOBAL LEADERS studies (81, 82). The MASTER-DAPT trial found a significant reduction in bleeding events in the 1 month DAPT arm whilst the GLOBAL LEADERS study did not (81). In contrast, the STOPDAPT-2 ACS trial which was recently presented found that clopidogrel monotherapy after 1 month of DAPT in an ACS population was associated with a higher risk of myocardial infarction even though a lower risk of bleeding was seen (83). Importantly, other than the GLOBAL LEADERS study, these were non-inferiority trials which were underpowered to evaluate ischemic events especially stent thrombosis. The GLOBAL LEADERS trial was designed as a superiority trial and did not demonstrate any benefit in terms of reduced bleeding risk, reduced all-cause mortality or non-fatal myocardial infarction with ticagrelor monotherapy.

The alternative option is to shorten the duration of dual antiplatelet therapy by early completion of P2Y_12_ inhibitor therapy. This has been investigated by multiple trials with variable durations of DAPT. The RESET (84), OPTIMIZE (85) and REDUCE (86) trials tested 3 months of DAPT compared to 12 months with ongoing aspirin after the defined DAPT period. Overall, these studies found no difference in the composite primary outcome that included bleeding and ischemic complications. These were also all non-inferiority trials that met their primary endpoint, however, concerns remain that they may be underpowered to evaluate ischemic outcomes such as stent thrombosis. The REDUCE trial illustrates this well where there was a non-significant but numerical increase in overall mortality (3.1 vs. 2.2%) and stent thrombosis (1.6 vs. 0.8%) in the 3-month compared to the 12-month DAPT group.

Several trials have also evaluated a 6-month duration of DAPT with subsequent aspirin compared to 12 months of DAPT. In the EXCELLENT trial, 6 months of DAPT was non-inferior to 12 months, however, the non-inferiority margin was quite wide for all-cause mortality and likely underpowered for this endpoint (87). The SECURITY, ISAR_SAFE, I-LOVE-IT 2, and SMART-DATE trials also suggested that 6 months of DAPT was not-inferior to 12 months (88–91). A meta-analysis of trials evaluating shorter durations of DAPT identified a reduced risk of major bleeding overall without an increase in ischemic events and death (92). However, as mentioned in the individual trials, the sample sizes are likely underpowered to evaluate ischemic outcomes.

Importantly, P2Y_12_ monotherapy compared to aspirin monotherapy post PCI has not been evaluated in a post ACS setting, including when direct oral anticoagulants (DOAC) are prescribed for concurrent AF. These are important areas for future research. Currently in ACS, 12 months of DAPT is recommended by both the ESC and ACC guidelines although shorter (6 months) or longer (greater than 12 months) durations can be considered for patients with high bleeding risk or a higher risk of recurrent ischemic events such as previous stent thrombosis, respectively (93).

The dosing of prasugrel and ticagrelor is another area for optimization in terms of potentially reducing bleeding risk. In the ISAR-REACT 5 trial, patients over the age of 75 years with a body weight less than 60 kg were prescribed 5 mg of prasugrel daily rather than 10 mg as a maintenance dose. This led to overall bleeding rates that were comparable between ticagrelor and prasugrel at 5.4 and 4.8%, respectively (94). A 60 mg twice daily ticagrelor maintenance dose has also been compared to the standard 90 mg twice daily dose in the PEGASUS-TIMI-54 trial for patients with a myocardial infarction in the preceding 1–3 years. With long-term DAPT, ticagrelor significantly reduced rates of the composite outcome of cardiovascular death, myocardial infarction or stroke at a median follow-up of 3 years. Importantly, at the lower 60 mg twice daily dose, rates of TIMI major bleeding were modestly reduced compared to standard maintenance dose. Both doses significantly reduced ischemic events compared to aspirin alone. Furthermore, in patients over 75 years of age, 5 mg prasugrel daily achieved similar inhibition of platelet activation as 10 mg daily, but with lower rates of mild bleeding (95). Prasugrel dose de-escalation may be another viable option to reduce bleeding risk with a reduction to 5 mg daily, 1 month after PCI. This may provide protection against secondary ischemic events whilst lowering the bleeding risk (96).

Future studies evaluating this may assist in determining the optimal regimen including dose of antiplatelet therapy for individual patients.

Vorapaxar, an inhibitor of thrombin mediated platelet activation *via* protease-activated receptor (PAR) 1 offers an alternative approach to reducing secondary ischemic events. The TRACER trial evaluated whether vorapaxar in addition to standard dual antiplatelet therapy is superior to placebo in reducing recurrent ischemic events post NSTEMI (97). Whilst the primary composite efficacy endpoint at 2 years was not significantly different between the groups, vorapaxar significantly reduced a composite of cardiovascular death, MI or stroke. However, this is offset by a higher bleeding risk. Of additional interest is the platelet function substudy of TRACER. It implies, based on the VASP assay, that concurrent vorapaxar administration may have a synergistic effect in increasing P2Y_12_ receptor inhibition; although this requires further dedicated research (98). Additionally, several *post hoc* analyses were undertaken which imply a potential benefit of vorapaxar in patients with low risk of bleeding and high risk of future ischemic events. The increased risk of bleeding with vorapaxar is certainly of concern, however, and its use is contraindicated in patients with a history of stroke, TIA or intracranial hemorrhage. Vorapaxar has also only been studied in combination with aspirin and/or clopidogrel and has not been rigorously tested with prasugrel or ticagrelor. Future trials will have to define the role of vorapaxar in this setting, although vorapaxar’s narrow ischemic benefit/bleeding risk profile may not be supportive of further clinical trial funding.

Concurrent atrial fibrillation (AF) in patients requiring P2Y_12_ inhibitor therapy also poses a conundrum in balancing bleeding risk with the combination of DOACs to prevent cerebrovascular events. The WOEST trial provided initial guidance when combining warfarin for atrial fibrillation with clopidogrel in patients post PCI or ACS. This suggested that the avoidance of triple therapy by omitting aspirin was associated with a reduction in bleeding complications but no excess risk of ischemic events at 1 year follow-up, although it was not adequately powered to evaluate stent thrombosis (99). Subsequently, multiple trials have demonstrated that DOACs can be combined with clopidogrel as dual therapy to effectively balance bleeding risk whilst still providing adequate protection against ischemic coronary complications as well as adequate protection against embolic cerebrovascular events due to AF (100–103).

Importantly when combined with clopidogrel, attention needs to be placed on the dosing of the individual DOACs. A lower dose than recommended for AF was studied for rivaroxaban (15 mg daily) and appeared to be the optimal dose for dabigatran (110 mg twice daily) at reducing bleeding risk (100, 101). In contrast, in the AUGUSTUS and ENTRUST-AF PCI trials, the recommended AF dosing of apixaban and edoxaban, respectively, were combined with clopidogrel and found to be an effective strategy at balancing bleeding and ischemic risk (102). In comparison, the addition of aspirin was generally associated with a significantly higher bleeding risk and should be reserved for patients at a high risk of ischemic coronary complications such as those with prior stent thrombosis (104). It should also be acknowledged, that whilst data is limited due to small numbers in the above DOAC trials, the combination of the more potent P2Y_12_ inhibitors, prasugrel and ticagrelor, are generally discouraged with DOACs due to the higher bleeding risk without significant demonstrable ischemic benefit (105). Finally, 12 months after PCI or ACS, single agent DOAC in patients with AF is the current recommendation without the need for long-term concurrent antiplatelet therapy (106).

## Future Directions

Future research toward optimizing antiplatelet therapy continues to focus on reducing bleeding risk that seems inherently linked with antiplatelet potency. One potential approach is the development of reversal agents, such as the monoclonal antibody bentracimab directed against ticagrelor. This development was heralded as one of the major advantages of ticagrelor and realized in the preliminary findings of the REVERSE-IT trial presented at the annual American Heart Association 2021 meeting. This recombinant IgG1 monoclonal neutralizing antibody has high affinity binding to ticagrelor and its major active metabolite (AR-C124910XX).

REVERSE-IT was a single arm phase III trial of 150 patients with major bleeding or requiring urgent surgery with ticagrelor administration within the preceding 72 h. A total dose of 18 g of bentracimab given over 12 h with initial bolus and loading doses was effective in reversing the antiplatelet effects of ticagrelor, as determined using the VerifyNow platelet function test and the VASP assay, the latter evaluating P2Y_12_ receptor inhibition. Furthermore, no rebound increase in platelet activity was seen after the use of the ticagrelor-neutralizing antibody bentracimab. Reversal was rapid within 5 min of bolus dose and maintained for 24 h. A prior trial in healthy volunteers reported similar findings and bentracimab was well tolerated with mainly minor infusion site adverse effects but no drug toxicity (107).

This provides an important tool in the armament, particularly for patients requiring urgent coronary bypass surgery in bail out situations or who are preloaded with ticagrelor before defining the coronary anatomy. It is also likely to play a role in patients with life-threatening bleeding where it is expected to achieve faster and more complete reversal of ticagrelor than platelet transfusion. Future trials demonstrating reduced bleeding complications compared to platelet transfusion without an increase in ischemic complications are eagerly awaited.

Another exciting future direction is the development of selatogrel, a novel subcutaneous, direct acting, and reversible P2Y_12_ inhibitor. This drug promises several advantages over existing oral P2Y_12_ inhibitors including a rapid onset of action and avoiding the vulnerabilities related to gastrointestinal absorption and hepatic metabolism (108, 109).

Additionally, the intermediate duration of action provides an exciting pre-loading strategy for further investigation, whereby selatogrel could be administered upstream prior to coronary angiography in ACS that would not compromise semi-urgent coronary bypass (if required) given its 24 h duration of action. This may provide the best of both worlds with rapid, near complete platelet inhibition at the time of PCI to reduce ischemic events with earlier offset than oral P2Y_12_ inhibitors to reduce bleeding complications if not required. It may also provide effective bridging antiplatelet activity until oral P2Y_12_ inhibitors reached therapeutic antiplatelet effect. Given that an infusion is not required, this could even be administered in the prehospital setting by emergency medical services and would be an interesting area of future research.

Inhibition of P2Y_12_ receptor-mediated platelet activation can be achieved *via* a promising alternative approach, the degradation of the respective agonist ADP. The endogenous NTPDase1, CD39, metabolizes ADP to adenosine monophosphate (AMP) which is then further metabolized to adenosine, itself exhibiting antiplatelet effects. Targeting CD39 to activated platelets *via* a single-chain antibody specific for the activated GPIIb/IIIa receptor accumulates antiplatelet effects selectively to forming clots (110). In preclinical data this approach has provided strong antithrombotic effects without bleeding time prolongation (110) and has also successfully been used in murine models of myocardial infarction (111).

## Conclusion

The platelet P2Y_12_ receptor remains a key therapeutic target and its antagonism comprises a cornerstone in the management of acute coronary syndromes. Significant investment has led to progressive improvement in drug design to address the limitations of the previous generations, leading to increased efficacy and a more consistent antiplatelet response. However, the trade-off between reducing ischemic events at the cost of increasing bleeding risk persists, which currently before new antiplatelet drugs enter the clinic can only be mitigated by individualized therapy. We look forward to novel therapeutics and patient-focused decision making that will provide clinicians with a greater armamentarium of drugs that allow individualized therapy tailored toward the individual patient’s risk of ischemic or bleeding events.

## Author Contributions

HF, JM, and KP were involved in manuscript writing. JM, XW, JS, DS, and KP critically reviewed the present manuscript. HF and KP were responsible for the overall content as guarantors. All authors contributed to the article and approved the submitted version.

## Conflict of Interest

The authors declare that the research was conducted in the absence of any commercial or financial relationships that could be construed as a potential conflict of interest.

## Publisher’s Note

All claims expressed in this article are solely those of the authors and do not necessarily represent those of their affiliated organizations, or those of the publisher, the editors and the reviewers. Any product that may be evaluated in this article, or claim that may be made by its manufacturer, is not guaranteed or endorsed by the publisher.
